# Orthopädie der Zukunft: KI trifft Bewegungsanalyse: Chancen und Risiken

**DOI:** 10.1007/s00132-025-04708-6

**Published:** 2025-08-26

**Authors:** Falko Heitzer, Dominik Raab, Wojciech Kowalczyk, Marcus Jäger

**Affiliations:** 1 Lehrstuhl für Orthopädie und Unfallchirurgie, Universität Duisburg-Essen, Kaiserstraße 50, 45468 Mülheim an der Ruhr, Deutschland; 2 St. Marien-Hospital Mülheim an der Ruhr, Klinik für Orthopädie, Unfall- und Wiederherstellungschirurgie, Mülheim an der Ruhr, Deutschland; 3 Lehrstuhl für Mechanik und Robotik, Universität Duisburg-Essen, Duisburg, Deutschland; 4 Philippusstift Essen, Klinik für Orthopädie, Unfall- und Wiederherstellungschirurgie, Essen, Deutschland

**Keywords:** Künstliche Intelligenz, Digitale Gesundheitstechnologien, Ganganalyse, Biomechanik, Muskuloskelettale Erkrankungen, Artificial intelligence, Digital health technology, Gait analysis, Biomechanics, Musculoskeletal diseases

## Abstract

**Hintergrund:**

Mit dem Voranschreiten der Digitalisierung sowie der rasanten Entwicklung portabler Messsysteme, ist die Integration neuer Technologien in die klinische Praxis unausweichlich. Unter Berücksichtigung des demographischen Wandels benötigt das derzeitige Gesundheitssystem genau diese Integration, um den wachsenden Herausforderungen angemessen zu begegnen. Im Hinblick auf die orthopädische Patientenversorgung repräsentiert die objektive Analyse des Bewegungsapparates einen immer wichtigeren Aspekt. Um dem Ausmaß instrumenteller Daten Herr zu werden und neue Chancen zu nutzen, werden innovative Schlüsseltechnologien benötigt. Während eine enorme Steigerung von Effizienz, Effektivität und Versorgungsqualität realisiert werden kann, dürfen mögliche Risiken durch die Anwendung von künstlicher Intelligenz (KI) nicht außer Acht gelassen werden.

**Methodik:**

Anhand einer Literaturrecherche nach der PRISMA-Methode werden spezifische Chancen für die klinische Integration identifiziert und etwaige Risiken benannt.

**Ergebnisse:**

Insgesamt wurden 190 Veröffentlichungen erfasst, von denen 50 zur näheren Betrachtung herangezogen wurden.

**Diskussion:**

Während die Anwendung von KI-Methoden zur Nutzung objektiver Messsysteme eine Vielzahl von Chancen in der orthopädischen Patientenversorgung eröffnet, gehen hiermit ebenfalls relevante Risiken einher. Um sowohl spezifische Problemstellungen zu behandeln als auch das übergeordnete Ziel der optimierten Gesundheitsversorgung zu erreichen, ist der bedarfsgerechte Einsatz und die richtige Integration von KI-Methoden unabdingbar. Hierbei ist zu erkennen, dass künstliche Intelligenz lediglich eine unterstützende Tätigkeit übernehmen kann, wobei Transparenz das Hauptkriterium für den vertrauensvollen Einsatz im Gesundheitswesen darstellt.

## Abstract (en)

**Background:**

With the progress of digitalization and the rapid development of portable measurement systems, the integration of new technologies into clinical practice is inevitable. Considering the demographic change, the current healthcare system needs exactly this integration to appropriately address the growing challenges. Regarding orthopaedic patient care, the objective analysis of the musculoskeletal system represents an increasingly important aspect. Innovative key technologies are necessary to handle the amount of instrumental data and create new opportunities. While an enormous increase in efficiency, effectiveness and quality of care can be realized, potential risks from the application of artificial intelligence (AI) need to be considered.

**Methods:**

Based on a literature review using the PRISMA-method, specific opportunities for clinical integration are identified and potential risks are discussed.

**Results:**

A total of 188 publications were included, of which 54 were analyzed in detail.

**Conclusion:**

While the application of AI methods for the use of objective measurement systems opens a variety of opportunities in orthopaedic patient care, it is also associated with relevant risks. In order to address specific problems and achieve the primary goal of optimized healthcare, the appropriate use and the proper integration of AI methods are essential. It must be acknowledged that artificial intelligence can only play a supporting role, with transparency being the main criterion for trustworthy use in the healthcare sector.

Der Einsatz von Methoden der künstlichen Intelligenz (KI) ist aktuell ein zentrales Thema im Gesundheitssektor und gilt in Kombination mit innovativer Messtechnik als zukunftsweisend, um durch Steigerung von Effizienz, Effektivität und Versorgungsqualität den Herausforderungen des demographischen Wandels zu begegnen. Speziell für die Nutzung von instrumentellen Bewegungsdaten in der orthopädischen Patientenversorgung repräsentiert KI eine vielversprechende Schlüsseltechnologie. Um eine einwandfreie, klinische Integration zu ermöglichen, müssen mögliche Chancen und Risiken durch den Einsatz von KI im medizinischen Kontext explizit berücksichtigt werden.

## Hintergrund und Fragestellung

Sowohl der gegenwärtige demographische Wandel als auch die rasante Entwicklung neuartiger, innovativer Messsysteme stellen das Gesundheitssystem vor neue und akute Herausforderungen [23, 33, 45]. Die stetig wachsende Anzahl hilfsbedürftiger Patienten mit Erkrankungen des Stütz- und Bewegungsorgans erfordert eine bedarfsgerechte Anpassung der Gesundheitsversorgung [54]. Unter Berücksichtigung des steigenden Renteneintrittsalters wird die Arbeitsfähigkeit auch im fortgeschrittenen Lebensalter vorausgesetzt [19, 28]. Parallel hierzu soll mit der zunehmenden Ambulantisierung von orthopädischen Eingriffen sowie einer Verkürzung der akut-stationären Verweildauer eine bedarfsgerechte Umstrukturierung gefördert werden. Fast-Track-Konzepte sollen Patienten bereits kurz vor operativen Eingriffen vorbereiten, die Eigenverantwortung fördern und eine Beschleunigung der Rehabilitation herbeiführen [29, 54]. Die hieraus resultierende, vermehrte Beanspruchung von Gesundheitsdienstleistungen erfordert gleichzeitig eine Entlastung aller beteiligten Arbeitsgruppen, um die Aufrechterhaltung einer hohen Versorgungsqualität zu gewährleisten.

Betrachtet man in der Orthopädie die Patientenversorgung im Allgemeinen, basiert Diagnosestellung, Behandlungsplanung und Rehabilitationssteuerung zumeist auf statischen Untersuchungen, einer Bildgebung mit Strahlenexposition sowie subjektiven Einschätzungen oder Assessment-Methoden [13]. Im Hinblick auf das Ziel einer zeitnahen Wiederherstellung der Funktion des Bewegungsapparates und dem damit verbunden schmerzfreien Alltag, stoßen die etablierten Methoden unter dem oben angeführten Szenario an ihre Grenzen. Hinzu kommt, dass der digitale Wandel in der Orthopädie zwar längst begonnen hat, jedoch neue Technologien noch nicht ausreichend in die klinische Praxis integriert sind. Innovative Systeme und optimierte Behandlungskonzepte haben das Potenzial, die medizinische Versorgung zu verbessern und eine Entlastung der beteiligten Arbeitsgruppen zu ermöglichen. Die praxistaugliche Implementierung und der zielspezifische Einsatz innovativer Messtechnologien repräsentieren gegenwärtige Herausforderungen bei der Versorgung orthopädischer Patienten [23, 45, 49].

Die vermehrte Nutzung instrumenteller Bewegungsdaten wird in Zukunft für die orthopädische Patientenversorgung unabdingbar sein. In den Bereichen Diagnostik, Behandlungsplanung, Rehabilitationsmonitoring und -steuerung verspricht der Einsatz innovativer Messsysteme und tragbarer Sensoren (Wearables) neue Möglichkeiten einer verbesserten Gesundheitsversorgung [2, 27, 44–46]. Eine Vielzahl unterschiedlicher Sensoren und Messsysteme ermöglichen eine portable Analyse von Bewegungen im Zuge der klinischen Untersuchung oder sogar im Alltag des Patienten [45, 50]. Diese Systeme produzieren jedoch hoch komplexe und multidimensionale Messdaten, deren Interpretation ein eigenes Fachgebiet darstellt [35, 36]. Deshalb ist die richtige Integration der Technologien maßgeblich für eine zielführende und bedarfsgerechte Nutzung [23]. Der klinische Einsatz solcher Messsysteme erfordert eine angemessene Verteilung zwischen Messgüte, Systemkosten, vorausgesetzter Nutzerqualifikation und benötigtem Zeitaufwand [53]. Dies ist vergleichbar mit der Anwendung tragbarer Sonographiegeräte, welche vermehrt in der Diagnostik eingesetzt werden [5]. Solche tragbaren Systeme müssen einfach und schnell anzuwenden sein, sodass keine Verlängerung der Untersuchungszeit resultiert. Gleichzeitig müssen die erhobenen Daten für das medizinische Personal direkt und verständlich auswertbar sein, sodass der Zeitaufwand hierfür minimal bleibt und keine zusätzliche Ausbildung erforderlich ist. Derzeitig werden in der Bewegungsanalyse, die durch instrumentelle Messsysteme produzierten Messdaten lediglich zur Auswertung einfacher Parameter (z. B. Schrittzahl, Ganggeschwindigkeit, Schrittlänge) verwendet [4, 35, 45].

Nicht nur der technische Fortschritt, sondern auch neue Untersuchungs- und Analysemethoden haben die Biomechanik allgemein und speziell die Betrachtung des menschlichen Bewegungsapparates stark beeinflusst [50, 53]. Besonders in der Bewegungsanalyse stellt die präzise und umfassende Datenanalyse ein bekanntes Problem dar [35, 37]. Bereits die Analyse multidimensionaler Datensätze von einzelnen Patienten repräsentiert sowohl für das medizinische Personal als auch für erfahrene Bewegungsanalytiker eine enorme Herausforderung. Sollen ganze Kollektive oder spezifische Patientengruppen untersucht werden, ist eine umfangreiche und qualitative Analyse selbst durch Spezialisten nicht mehr manuell umsetzbar. Der Informationsgehalt, welcher derartigen Daten entzogen werden kann, kann durch herkömmliche Analysen nicht vollumfänglich genutzt werden [4, 23, 28].

Dies verdeutlicht, dass die digitale Transformation des Gesundheitswesens nicht nur neuartige oder miniaturisierte Sensoren benötigt, sondern auch Technologien, welche es erst ermöglichen, die technischen Fortschritte in der Praxis gewinnbringend einzusetzen. Der dafür erfolgversprechendste Ansatz ist der richtige Einsatz von künstlicher Intelligenz (KI) [15, 28]. Unter Berücksichtigung der in den letzten Jahren entstanden Möglichkeiten zum Einsatz von KI-Methoden, besteht ein deutlicher Rückstand bei der klinischen Anwendung solcher Schlüsseltechnologien in Kombination mit innovativer Messtechnologie [45, 53].

Daher gilt es, die Entwicklung von KI-Anwendungen in Kombination mit praxistauglichen Messsystemen für die klinische Anwendung zu fokussieren und eine gezielte Integration in die orthopädische Patientenversorgung zu ermöglichen. Hierzu werden im Folgenden die wesentlichen Chancen und potenziellen Risiken für den Einsatz von KI im klinischen Kontext identifiziert und anschließend diskutiert.

## Methodik

Zur Bewertung der Chancen und Risiken beim Einsatz von KI für die Analyse von Bewegungsdaten in der orthopädischen Patientenversorgung wurde eine systematische Literaturrecherche ausschließlich in der Datenbank Google Scholar durchgeführt. Unter Verwendung der PRISMA-Methode [26] wurde mittels dem nachfolgenden Suchstring (Schlüsselbegriffe in der Suchabfrage auf Deutsch) nach allen Veröffentlichungen bis 2025 (erstes Quartal) gesucht:

*Ganganalyse AND „künstliche Intelligenz“ OR Bewegungsanalyse AND „künstliche Intelligenz“*

**Abb. 1:**
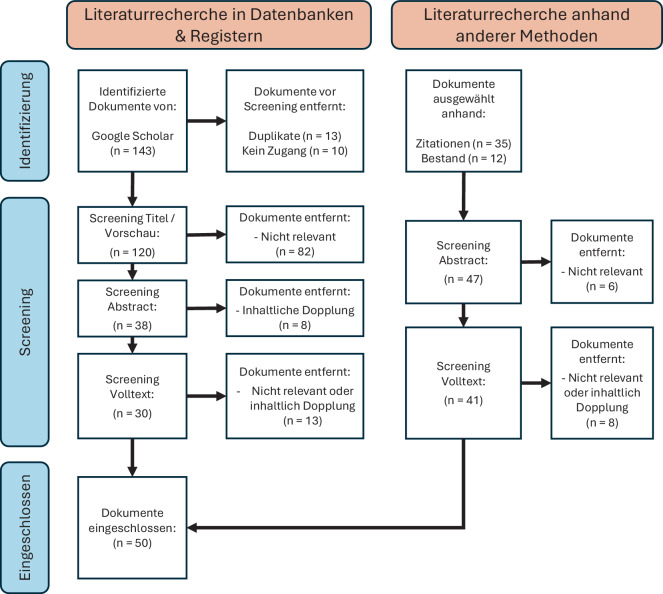
Flussdiagramm der Literaturrecherche

Um das Thema KI in der Bewegungsanalyse umfassend zu untersuchen und ein narratives Review zu verfassen, wurden im Kontext der Literaturrecherche sowohl fachspezifische Arbeiten als auch allgemeine Literatur zur KI in der Medizin berücksichtigt. Wie in Abb. 1 dargestellt ist, wurden die in Google Scholar und mittels anderer Methoden gefundenen Dokumente in einem mehrstufigen Prozess gescreent. Hierbei wurde mit dem Fokus auf die Chancen bei der Anwendung von KI-Methoden im orthopädischen Sektor, sowie möglichen Folgen und Risiken eine Auswahl an relevanten Veröffentlichungen getroffen.

## Ergebnisse

Im Rahmen der Literaturrecherche wurden 190 relevante Veröffentlichungen (Datenbank 143, andere Methoden 47) identifiziert, von denen 50 Veröffentlichungen zur weiteren Analyse eingeschlossen wurden. Tab. 1 zeigt alle nach dem Screening eingeschlossenen Veröffentlichungen mit Quellenangabe und zugehöriger Zitatnummer in alphabetischer Reihenfolge. Nachfolgend werden mögliche Chancen für den klinischen Einsatz sowie eventuelle Risiken durch die Anwendung von KI aus den Volltexten der identifizierten Literatur herausgearbeitet. Im Zuge des Volltexte-Screenings werden Textstellen und Informationen zur Entwicklung, Integration, Applikation und Bewertung möglicher Auswirkungen und Folgen durch KI allgemein und speziell im klinischen Szenario betrachtet.

**Tab. 1:** Eingeschlossene Veröffentlichungen nach Screening

Nr.	Quelle
[1]	Begg R, Kamruzzaman J (2005), 10.1016/j.jbiomech.2004.05.002
[2]	Brand A et al. (2024), 10.1007/s00132-024-04516-4
[3]	Dindorf C et al. (2023), 10.1007/978-3-662-67419-2
[4]	Dindorf C et al. (2024), 10.1007/978-3-031-67256-9
[6]	Fricke L et al. (2024), 10.1007/s00142-024-00667-w
[7]	Fricke L et al. (2024), 10.1016/j.orthtr.2024.04.002
[8]	Fröhlich M et al. (2025), 10.22028/D291-44473
[9]	Garcia-Agundez A, Eickhoff C (2025), 10.1007/s00398-024-00664-z
[10]	Gethmann CF et al. (2022), 10.1007/978-3-662-63449-3
[11]	Gondlach K et al. (2024) 10.1007/978-3-658-44852-3
[12]	Greco M et al. (2023), 10.3390/diagnostics13040630
[14]	Hiltawsky K, Boll S (2022), 10.48669/pls_2022-3
[15]	Holm J (2017), 10.5169/seals-736588
[16]	Hornegger J (2021), 10.1007/978-3-658-35779-5_33
[17]	Jahn K (2022), 10.1055/a-1822-3227
[18]	Jakubowitz E et al. (2023), 10.3390/app13095728
[19]	Jansen D (2021), https://digitaleweltmagazin.de/d/magazin/DW_21_03.pdf. Zugriff: 19.03.2025
[20]	Jedamzik S (2019), 10.1007/s10405-019-00279-4
[21]	Kirchner EA et al. (2016), ISBN: 978-3-86818-090‑9
[22]	Knappertsbusch I, Gondlach K (Hrsg) (2021), 10.1007/978-3-658-35779-5
[23]	Kuhn S, Knitza J (2024), 10.1007/s00132-024-04496-5
[24]	Lebleu J et al. (2021), 10.1016/j.bjpt.2019.12.002
[25]	Matzka S (2021), 10.1007/978-3-658-34641-6
[27]	Moon Y et al. (2017), 10.1371/journal.pone.0171346
[28]	Mundt M et al. (2020), 10.1007/978-3-662-58474-3
[29]	Nöth U et al. (2025), 10.1007/s00132-025-04617-8
[32]	Paass G, Hecker D (2020), 10.1007/978-3-658-30211-5
[33]	Pfannstiel MA (2022), 10.1007/978-3-658-33597-7
[34]	Pradhan C et al. (2015), 10.1016/j.jelekin.2015.01.004
[35]	Raab D, Kecskeméthy A (2023), 10.1007/s00132-023-04397-z
[36]	Raab D et al. (2023), 10.1007/s00264-022-05670-0
[37]	Raab D et al. (2025), 10.1016/j.gaitpost.2025.01.072
[38]	Reumann MK et al. (2022), 10.1007/s00113-022-01202-y
[39]	Rezapour M et al. (2024), 10.1002/jor.25837
[40]	Rubeis G (2024), 10.1007/s00740-024-00539-x
[41]	Rüping S, Sander J (2018), 10.1007/978-3-662-57611-3_2
[42]	Samhammer D (2023), 10.1007/978-3-662-67008-8
[43]	Schmailzl KJG (2021), 10.1007/s00129-021-04815-3
[44]	Smith VM et al. (2018), 10.1177/2055217317753465
[45]	Smits Serena R et al. (2024), 10.1007/s00132-024-04567-7
[46]	Syversen A et al. (2024), 10.3390/s24020482
[47]	Tretter M et al. (2024), 10.1007/s00481-023-00789-z
[49]	Weidemann ML et al. (2019), 10.1007/s00115-019-00817-8
[50]	Welke B, Jakubowitz E (2025), 10.1007/s00113-025-01549-y
[51]	Wennker P (2020), 10.1007/978-3-658-30480-5
[52]	Willwacher S, Korn O (2021), 10.1007/978-3-030-80829-7_104
[53]	Willwacher S et al. (2023), 10.1007/s00132-023-04404-3
[55]	Youssef Y et al. (2024), 10.1007/978-3-662-70070-9
[56]	Zago M et al. (2020), 10.3389/fbioe.2020.638793
[57]	Zimmer Biomet (2025), https://www.zimmerbiomet.com/en/patients-caregivers/mymobility-patient-care-app.html. Zugriff: 25.04.2025

### Chancen durch KI-Einsatz in der Medizin

**Abb. 2:**
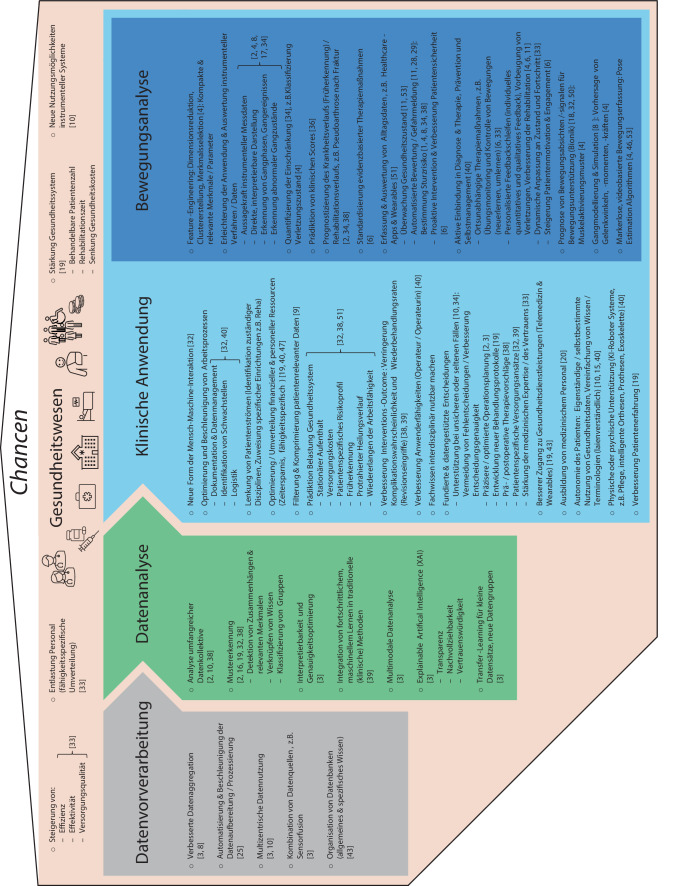
Chancen durch den Einsatz von KI im Gesundheitswesen

Die Nutzung instrumenteller Messdaten durch KI-Methoden repräsentiert eine vielversprechende Möglichkeit, um in allen Bereichen der Gesundheitsversorgung eine Steigerung von Effizienz, Effektivität und Versorgungsqualität zu erreichen [33]. Betrachtet man den Einsatz von KI in der orthopädische Patientenversorgung, lassen sich relevante Chancen in den folgenden, voneinander abhängigen Bereichen identifizieren: Datenvorverarbeitung, Datenanalyse, klinische Anwendung, Bewegungsanalyse (Abb. 2). Die Anwendung von KI-Methoden in den einzelnen Bereichen trägt jeweils zum übergeordneten Ziel bei, das Gesundheitssystem zu entlasten und die Gesundheitsversorgung zu verbessern [19].

Bereits während der **Datenvorverarbeitung** kann KI den Anwender unterstützen. Messfehler können detektiert und korrigiert, unterschiedliche Datenquellen kombiniert und Daten aus verschiedenen Zentren nutzbar gemacht werden [3, 8, 10, 25, 43].

Der zentrale Aspekt bei der Nutzung von KI-Methoden ist jedoch die **Datenanalyse**. Dort, wo die Analysefähigkeit des Menschen an ihre kapazitiven Grenzen stößt, kann KI unterstützen und neue Möglichkeiten für die Auswertung hoch komplexer Daten schaffen [2, 10, 38]. Mustererkennung bei multimodalen Daten, Verknüpfung von Wissen aus unterschiedlichen Bereichen und die Analyse umfangreicher Patientenkollektive sind durch spezifische KI-Methoden realisierbar [2, 3, 16, 19, 32, 38]. Für den klinischen Einsatz besonders wichtig sind Methoden, welche in traditionelle klinische Prozesse integriert werden können, wodurch eine Optimierung der Behandlung erreicht werden kann [3, 39]. Hierbei spielt die erklärbare künstliche Intelligenz eine besondere Rolle („*explainable artificial intelligence*“ [XAI]), um die Nachvollziehbarkeit der KI-gestützten Datenanalyse zu gewährleisten und die Ergebnisse vertrauenswürdig sowie transparent darzustellen [3].

In der **klinischen Anwendung** kann KI zahlreiche Hintergrundprozesse optimieren und Belastungszustände vorhersagen (z. B. Versorgungkosten, Risikoprofile, Heilungsverläufe) [32, 38, 40, 51]. Speziell für die Orthopädie stellen die Verbesserung des Interventions-Outcomes und die Rehabilitationssteuerung zentrale Aspekte bei der Patientenversorgung dar [38, 39]. Durch die Unterstützung von KI können umfangreichere Patientendaten einbezogen und somit klinische Entscheidungen fundiert und datengestützt getroffen werden [9]. Schwierige Fälle können mithilfe von Unterstützungssystemen besser behandelt werden, wodurch eine Verringerung der Komplikationswahrscheinlichkeit und Wiederbehandlungsrate erzielt werden kann [10, 34, 55]. Mit Blick auf die direkte Interaktion zwischen Patient und KI-System, fördert die Anwendung im medizinischen Bereich die Autonomie des Patienten, welcher selbstbestimmt viele seiner Gesundheitsdaten laienverständlich auslesen und eigenständig nutzen kann [10, 15, 40]. Durch die Kombination von telemedizinischen Applikationen und tragbaren, smarten Sensoren mit KI erlangen sowohl der Patient als auch das ärztliche Personal einen differenzierten Einblick in den alltäglichen Gesundheitszustand [19, 43].

Besonders in der **Bewegungsanalyse** spielt dies eine essenzielle Rolle, da die präzise Erfassung des natürlichen Gangbildes bisher ein sehr umfangreiches Unterfangen darstellte. Anhand von Bewegungsdaten und einer spezifischen Analyse sowie Darstellung mittels KI, können sowohl patientenindividuelle Merkmale und Parameter identifiziert und zur Quantifizierung der Einschränkung des Bewegungsapparates genutzt werden [2, 4, 8, 17, 18, 34]. Aufbauend darauf können nicht nur Krankheitsverläufe prognostiziert, sondern auch spezifische Therapiekonzepte erstellt, standardisiert und dynamisch während der Rehabilitation angepasst werden [2, 6, 34, 38]. Ebenfalls ermöglichen KI-Methoden zusammen mit einer kontinuierlichen Datenerfassung im Alltag eine Früherkennung und Gefahrenmeldung bei abnormalen Zuständen oder auftretenden Ereignissen (z. B. Überwachung Gesundheitszustand, Bestimmung Sturzrisiko) [1, 11, 12, 28, 29, 51, 53]. Neben präventiven Maßnahmen kann der Patient mit der Unterstützung von KI-Systemen ebenfalls aktiv in die Nachsorge eingebunden werden, bei der personalisierte Feedbackschleifen den Patienten in der richtigen Übungsausführung unterstützen oder Schwierigkeitsgrade fortschrittsabhängig eingestellt werden [6, 11, 24, 33, 40, 45].

Übergeordnet erfährt das **Gesundheitswesen** eine weitreichende Verbesserung sowohl durch die direkte Anwendung von KI in der klinischen Praxis und Bewegungsanalyse als auch durch die Nutzung zur Datenvorverarbeitung und -analyse [50]. Neben der anfangs beschriebenen Steigerung der Versorgungsqualität kann medizinisches Personal entlastet oder fähigkeitsspezifisch umverteilt werden. Durch die Erhöhung der Anzahl an behandelbaren Patienten, eine Verringerung der Rehabilitationszeit sowie einer Senkung von Gesundheitskosten, kann das Gesundheitssystem gestärkt werden [19].

### Mögliche Risiken durch KI-Einsatz

**Abb. 3:**
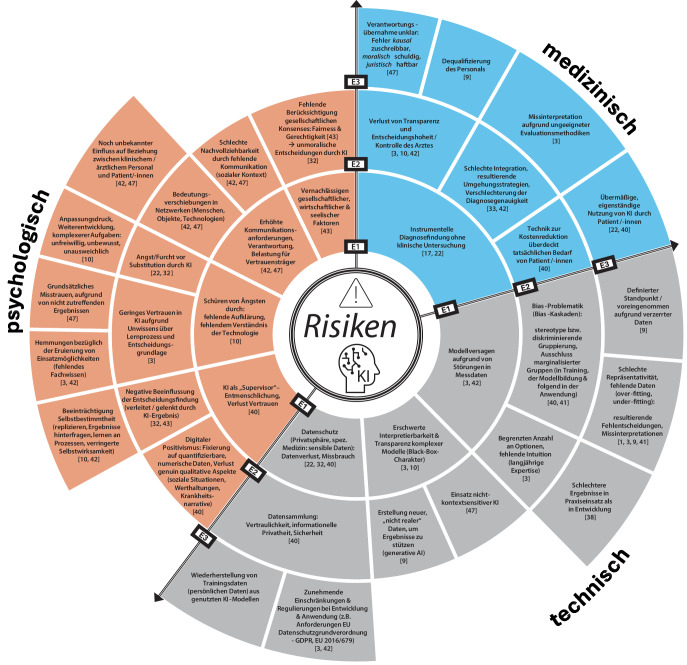
Risiken durch Künstliche Intelligenz im Gesundheitswesen

Etwaige Risiken durch den Einsatz von KI in der klinischen Praxis können in drei Hauptkategorien untergliedert werden: **technisch, medizinisch, psychologisch**. Die Abb. 3 veranschaulicht diese Untergliederung und hebt die Abhängigkeiten von einzelnen Risiken untereinander durch verschiedenen Ebenen (Ebene 1 bis Ebene 3) hervor. Auf der innersten Ebene (E1) werden zunächst die zentralen, übergeordneten Risiken dargestellt, auf denen die Risiken in den höheren Ebenen (E2 und E3) aufbauen und diese mit zusätzlichen Punkten spezifizieren.

#### Medizinische Risiken

Als zentrales medizinisches Risiko ist die instrumentelle Diagnosefindung ohne klinische Untersuchung zu sehen, welche bedingt durch die Weiterentwicklungen im Bereich Messsensorik und KI zu einer übermäßigen Nutzung führen kann [17, 22]. Auch wenn KI-gestützte Methoden lediglich als Zusatz zur klinischen Untersuchung verwendet werden, besteht die Möglichkeit des Verlustes von Transparenz und Entscheidungshoheit bzw. Kontrolle des Arztes [3, 10, 42]. Dieses Risiko kann durch die folgenden Fragen näher definiert werden: Inwieweit versteht das ärztliche Personal die Entscheidung des KI-Systems und kann diese nachvollziehen? Beruht die Entscheidung des ärztlichen Personals ausschließlich auf der Systemausgabe oder kann das ärztliche Personal Entscheidungen entgegen der Systemausgabe treffen? Bei der Nutzung von KI-Systemen in der klinischen Anwendung kann hierdurch eine Unklarheit in Bezug auf die Verantwortungsübernahme entstehen. Bereits vor dem Eintreten von etwaigen Fehlern muss für die Anwendung klar definiert sein, wem der Fehler kausal zuzuschreiben ist, wer hierbei moralisch schuldig ist und auch wer juristisch dafür haftbar gemacht werden kann [47]. Dies dient nicht nur zur Vermeidung eines möglichen Rechtsstreits, sondern auch zum Erhalt der ärztlichen Verantwortung in medizinischen Behandlungen und dem Entgegenwirken einer möglichen Dequalifizierung des Personals [9]. Betrachtet man bei den medizinischen Risiken den Patienten als Individuum, kann das Ziel der Kostenreduktion im Gesundheitswesen durch KI-Methoden schnell zu einer Überdeckung des eigentlichen Patientenbedarfs führen. Dies ist auch verknüpft mit der übermäßigen eigenständigen Nutzung von KI durch den Patienten selbst [22, 40].

#### Technische Risiken

Drei zentrale Aspekte können bei den technischen Risiken identifiziert werden. Diese betreffen die *Modellzuverlässigkeit*, die *Interpretierbarkeit* und den *Datenschutz*.

Ein Modellversagen, und resultierend daraus die Erzeugung von fehlerhaften Ergebnissen für die klinische Anwendung, wäre fatal und würde weitreichende Folgen für Patienten nach sich ziehen. Ein solches Versagen kann sowohl auf Trainings- als auch auf Anwendungsdaten beruhen [3, 42]. Eine Ursache hierbei kann die Bias-Problematik sowie mögliche Bias-Kaskaden sein. Stereotype und der Ausschluss marginalisierter Gruppen können schwerwiegende Folgen für alle Schritte der Bildung von KI-Modellen haben. Diese Fehler können sich im gesamten Prozess fortziehen und verstärken [40, 41].

Wie bereits in den medizinischen Risiken angeschnitten, ist das Verstehen des KI-Modells und dessen Ausgabe relevant für die Integration in die Praxis. Hierbei gilt es bereits auf technischer Seite möglichen Risiken, wie der erschwerten Interpretierbarkeit durch zu komplexe Modelle, mit einem „Black-Box-Charakter“ zu begegnen [3, 10]. Komplexe Modelle wie zum Beispiel generative KI-Modelle können zur Erstellung und Verwendung nicht realer Daten führen, was für den Anwender nicht direkt ersichtlich ist [9].

Besonders bei der Anwendung von KI auf sensible Daten stellen Datenverlust oder Missbrauch große Risiken dar. Vor dem Hintergrund des Modelltrainings oder der Modellanwendung sind ebenfalls größere Datensammlungen gefährdet [22, 32, 40].

#### Psychologische Risiken

Ein zentraler Aspekt hierbei ist die grundsätzliche Angst vor der Technologie „KI“, da das fehlende Verständnis oder eine fehlende Aufklärung zu einem falschen Bild der Technologie führen kann [10]. Ein häufiges Beispiel ist die Angst vor der Substitution durch KI im Beruf [22, 32]. Die teilweise unausweichliche, unfreiwillige oder unbewusste Nutzung von KI führt zu einem Anpassungsdruck auf den Anwender. Um neuen Standards gerecht zu werden und im Zuge der Weiterentwicklung mitzuhalten, muss das medizinische Personal die neue Technologie verstehen und gewinnbringend einsetzen können [10]. Das Verstehen der Technologie sowie der vorliegenden Lern- und Entscheidungsprozesse des KI-Systems sind der Grundstein für das Vertrauen und die Eruierung von Einsatzmöglichkeiten. Somit ist Unwissenheit ein wesentliches Risiko bei der Anwendung in der klinischen Praxis [3, 42]. Bedingt durch negative Erfahrungen besteht ein Risiko, dass dieses Vertrauen nachlässt. Wie bereits erwähnt, bieten auch computergestützte Systeme keine hundertprozentige Zuverlässigkeit, weshalb Ergebnisse durch den Nutzer validiert und der Lösungsweg nachvollzogen werden muss. Nur so können Systemfehler verstanden und eine mögliche fehlerhafte, medizinische Behandlung vermieden werden [47].

Als weiteres zentrales Risiko ist die Entmenschlichung in der medizinischen Behandlung anzuführen. Eine übermäßige Nutzung der KI als „Supervisor“ kann zum Verlust des Vertrauens in das medizinische Personal führen [40]. Dieser Prozess kann sowohl zu einer negativen Beeinflussung der Entscheidungsfindung bei allen beteiligten Personen führen [32, 43], als auch einen digitalen Positivismus auslösen. Der KI-Nutzer fixiert hierbei lediglich die quantifizierbaren, numerischen Daten (inhärenter Reduktionismus) und vernachlässigt die genuin qualitativen Aspekte (soziale Situationen, Werthaltungen, Krankheitsnarrative) [40]. Die vermehrte Beeinflussung des KI-Anwenders kann in nahezu allen Bereichen (Entwicklung, Forschung, Praxiseinsatz) zu einer Beeinträchtigung der Selbstbestimmtheit führen. Besonders im orthopädischen Bereich ist ein Verlust der fachspezifischen Fähigkeiten (z. B. Wissen replizieren, Ergebnisse hinterfragen, Lernen an Prozessen) kritisch zu sehen [10, 42]. Hierzu zählt ebenfalls die Fähigkeit, Situationen richtig in den bestehenden Kontext einzuordnen. Das Vernachlässigen von gesellschaftlichen, wirtschaftlichen oder seelischen Faktoren, resultierend aus dem Einsatz von KI, stellt ein relevantes Risiko dar [32, 43].

Auch wenn der Einsatz von KI in der klinischen Praxis eine Möglichkeit zur Entlastung bietet, muss ein für die Integration zentraler Aspekt berücksichtigt werden: Das medizinische Personal und vor Allem der ärztliche Anwender stehen in der Verantwortung einer erhöhten Kommunikationsanforderung gerecht zu werden. Mit der Nutzung von KI-Systemen gilt es, dem Patienten die Behandlungsentscheidung weitestgehend verständlich und den dahinterstehenden Entscheidungsprozess transparent offen zu legen. Dies kann zu einer erhöhten Belastung der Vertrauensträger führen, da nicht nur die eigene Expertise, sondern auch die verwendeten KI-Systeme dargestellt werden müssen. Besonders im sozialen Kontext birgt dies für Patienten und Angehörige das Risiko einer schlechteren Nachvollziehbarkeit, da ein grundlegendes Verständnis der benutzen Technologien nicht vorausgesetzt werden kann. Dies wird zu Bedeutungsverschiebungen in Netzwerken führen (Menschen – Objekte – Technologien), wodurch ein Wandel im Gesundheitswesen unabdingbar ist. Bisher können die Auswirkungen des Wandels und der Einfluss auf die Beziehungen zwischen medizinischem Personal und den Patienten noch nicht vollständig vorhergesagt werden [42, 47]. Daher sind die Entwicklung, Integration und finale Anwendung im klinischen Szenario mit äußerster Aufmerksamkeit und Umsicht durchzuführen.

## Diskussion

Der Einsatz von KI als Schlüsseltechnologie zur effektiven Nutzung instrumenteller Messdaten in der Orthopädie und Unfallchirurgie bietet eine vielversprechende Lösung, um derzeitige Herausforderungen zu bewältigen und das Gesundheitssystem ganzheitlich zu stärken. „Auch wenn KI-Systeme bereits in einigen Bereichen klinischer Praxis zur Entscheidungsunterstützung eingesetzt werden, liegt der Großteil ihres Potenzials noch in der Zukunft“ [42]. Hierbei darf der „unreflektierte, pauschale Einsatz der Methodik“ nicht der KI-Forschung vorangehen [3]. Auch wenn der Einsatz von KI ein enormes Ausmaß an Chancen in allen Bereichen und speziell in der instrumentellen Bewegungsanalyse bietet, müssen Auswirkungen und Einflussfaktoren umfassend erforscht werden. Im Hinblick auf eine optimale Integration in die klinische Praxis gilt es, alle relevanten Risiken zu thematisieren und klare Lösungsstrategien zu entwickeln.

Besonders wichtig ist hierbei die Transparenz von KI-Systemen, um Vertrauen in die Systemausgaben zu schaffen. „Nicht alle Nutzer:innen müssen das System im Ganzen verstehen, um ihm zu vertrauen, aber sie sollten wissen, warum sie ihm vertrauen können“ [42]. Sowohl das medizinische Personal als auch die behandelten Patienten sollten ein grundlegendes Verständnis der Technologie erlangen, wie es bei anderen Systemen im medizinischen Bereich der Fall ist (z. B. Bildgebung). Die Aufklärung über die spezifische Anwendung und die resultierenden Ergebnissen von KI-Systemen muss von den Vertrauensträgern (z. B. ärztliches Personal) übernommen werden, sodass auch die Verantwortungsfrage klar beantwortet werden kann. „Anforderungen an die Einführung neuer Systeme in den klinischen Alltag sind, partizipative Entscheidungsfindung zu fördern und Ansprüchen an Vertrauen, Transparenz und Verantwortungszuschreibung gerecht zu werden“ [42]. Dies beginnt bereits bei der Ausbildung und Weiterbildung von medizinischem Personal, welche an die Entwicklung des Gesundheitswesens und der Gesundheitsversorgung angepasst werden muss. „Ohne den Einbezug des medizinischen Personals wird es letztendlich schwierig sein, Vertrauen in neue KI-Systeme zu generieren“ [42]. Dies gilt insbesondere für die Orthopädie und Unfallchirurgie, die im Vergleich zu anderen Fachdisziplinen (z. B. Radiologie, Labormedizin) KI-Systeme noch verhältnismäßig selten in die klinischen Abläufe integriert haben.

Ein wesentlicher Punkt ist hierbei die Kommunikation, welche aufgrund der Bedeutungsverschiebung in den relevanten Netzwerken für die richtige Integration in die klinische Praxis essenziell ist [20, 32, 42, 47]. Es muss allen Beteiligten und vor allem den Patienten klar offengelegt werden, dass KI zur Entlastung des Personals und zur Verbesserung der Gesundheitsversorgung als unterstützendes Werkzeug eingesetzt wird, jedoch nicht die Verantwortung für wichtige Entscheidungen übernehmen kann [32]. „Denn selbst bei gut trainierten KI-Systemen werden auch in Zukunft noch Fehler auftreten und falsche Vorschläge gemacht werden“ [32].

„KI unterstützt, kann aber nicht ersetzten. Stattdessen ermöglicht KI schneller und präziser zu handeln“ [3]. Ob individualisierte Behandlungskonzepte, personalisierte Therapie, automatisierte Feedbackschleifen oder alltägliche Gefährdungsbewertung von Risikopatienten, die Möglichkeiten zum Einsatz von smarten Sensortechnologien im Bereich Bewegungsanalyse in Kombination mit KI-Methoden eröffnen weitreichende Chancen in der klinischen Praxis [28]. KI stellt hierbei nicht nur ein Werkzeug dar, sondern erlaubt es, die menschliche Bewegung auf eine neue Art und Weise zu betrachten [56]. Unter Berücksichtigung der Chancen und Risiken kann KI zielgerichtet in die Behandlung von orthopädischen Patienten integriert werden. Sowohl die Diagnostik als auch die Behandlungs- und Rehabilitationsplanung sind Anwendungsfelder, in denen die Einbindung von intelligenten, KI gestützten Systemen unausweichlich ist und die Zukunft der Orthopädie maßgeblich prägen wird.

## Fazit für die Praxis

- Die instrumentelle Bewegungsanalyse ist ein mächtiges Tool zur objektiven Beurteilung des Bewegungsapparates, welches aufgrund derzeitiger Entwicklungen im Bereich Wearables zunehmend an Bedeutung für die klinische Anwendung gewinnt.
- Um resultierende, komplexe Messdaten klinisch nutzbar und direkt interpretierbar zu machen, ist der Einsatz von Künstlicher Intelligenz (KI) notwendig.
- KI unter Beachtung relevanter Risiken repräsentiert eine Schlüsseltechnologie zur Optimierung der Gesundheitsversorgung.
- Technische Risiken müssen bereits vor Entwicklung durch eine interdisziplinäre Zusammenarbeit identifiziert werden.
- Medizinische Risiken entstehend aus der Anwendung von KI erfordern eine spezifische Integration sowie eine grundlegende Ausbildung des Personals.
- Psychologische Risiken können sowohl durch Aufklärung als auch durch eine transparente Kommunikation zwischen allen Beteiligten behandelt werden.
- KI als innovatives Werkzeug kann in der Gesundheitsversorgung unterstützen, jedoch nicht ersetzen.

## Abkürzungen

AI: Artificial intelligence
E: Ebene
EU: Europäische Union
GDPR: General data protection regulation
KI: Künstliche Intelligenz
PRISMA: Preferred reporting items for systematic reviews and meta-analyses
XAI: Explainable artificial intelligence
